# Determining factors associated with cholera disease in Ethiopia using Bayesian hierarchical modeling

**DOI:** 10.1186/s12889-022-14153-1

**Published:** 2022-09-20

**Authors:** Tsigereda Tilahun Letta, Denekew Bitew Belay, Endale Alemayehu Ali

**Affiliations:** 1 Department of Statistics, Ambo University, Ambo, Ethiopia; 2 Department of Statistics, Bahir Dar University, Bahir Dar, Ethiopia

**Keywords:** Cholera, Integrated Nested Laplace Approximation, Latent Gaussian model, Outbreak

## Abstract

**Background:**

Cholera is a diarrheal disease caused by infection of the intestine with the gram-negative bacteria Vibrio cholera. It is caused by the ingestion of food or water and infected all age groups. This study aimed at identifying risk factors associated with cholera disease in Ethiopia using the Bayesian hierarchical model.

**Methods:**

The study was conducted in Ethiopia across regions and this study used secondary data obtained from the Ethiopian public health institute. Latent Gaussian models were used in this study; which is a group of models that contains most statistical models used in practice. The posterior marginal distribution of the Latent Gaussian models with different priors is determined by R-Integrated Nested Laplace Approximation.

**Results:**

There were 2790 cholera patients in Ethiopia across the regions. There were 81.61% of patients are survived from cholera outbreak disease and the rest 18.39% have died. There was 39% variation across the region in Ethiopia. Latent Gaussian models including random and fixed effects with standard priors were the best model to fit the data based on deviance. The odds of surviving from cholera outbreak disease for inpatient status are 0.609 times less than the outpatient status.

**Conclusions:**

The authors conclude that the fitted latent Gaussian models indicate the predictor variables; admission status, aged between 15 and 44, another sick person in a family, dehydration status, oral rehydration salt, intravenous, and antibiotics were significantly associated with cholera outbreak disease.

**Supplementary Information:**

The online version contains supplementary material available at 10.1186/s12889-022-14153-1.

## Introduction

Cholera is an infectious disease characterized by large volumes of diarrhea and succeeding dehydration. It is an acute diarrheal infection caused by the digestion of food or water contaminated with the bacterium Vibrio cholera. It infected both children and adults, can kill within hours if left untreated [1].

Globally, in 2015 approximately 2.65 million new cases (range from 1.3 million to 4.0 million) and approximately 82,000 deaths (range from 21,000 to 143,000) every year have been occurred worldwide due to cholera [2].

In Africa from 15 countries; there are 120,652 cholera cases and 2436 deaths have occurred. The most estimated number of cholera cases are in West Africa around 40% cholera cases, in East Africa and Horn of Africa approximately 32% cholera cases, and 28% in central and middle Africa. The most death occurs the continent was central and middle Africa (43.4%), in West Africa approximately 37.5% of deaths occurred and the rest 19.1% occurred in East Africa and the Horn of Africa [3].

A different study reported from various regions about the cholera outbreak showed that the total cases ranged between 25 to 36,154 cholera cases and around 246 deaths in Ethiopia. This burden of the diseases was gradually increased from year to year [4, 5].

Though the infectious disease is quite serious due to rapid spread and has burdensome of death, only limited studies have been conducted in the world and specifically in Ethiopia. On the other hand, most of the studies conducted in Ethiopia were limited to some zones and maximum region [5–7]. Besides, those studies were more descriptive based for which they were not properly addressing the basic research questions. Some of those studies used a case–control method that there were not going through the assumptions of the models they applied. Hence, the collective reasons stated above and a rare study conducted, the researcher tried to fill the gap by using appropriate statistical models and assess the risk factors of cholera outbreak in Ethiopia.

## Methods

### Study area

Ethiopia is the oldest independent country in Africa. It is located in the center of the Horn of Africa. The country covers an area of 1,126,829 square kilometers. Ethiopia is a Federal Democratic Republic composed of 9 National Regional states: namely Tigray, Afar, Amhara, Oromia, Somali, Benishangul-Gumuz, Southern Nations Nationalities, and Peoples’ Region (SNNPR), Gambella and Harari, and two Administrative states Addis Ababa City administration and Dire Dawa city council.

### Data and variables

The data for this study was secondary and it is obtained from Ethiopian Public Health Institute (EPHI). It is reported from different regional health offices and the two administrative cities in the study period from April 2019 to January 2020 used for this study.

The inclusion criterion of this study was all cholera outbreak patients in all age groups at Addis Ababa, Afar, Amhara, Harari, Oromia, SNNPR, Somali, and Tigray from April 2019 to January 2020. There were no cholera cases reported from regions like Benishangul-Gumuz, Gambella, and Dire Dawa administrative city during the data collection period, and these regions are not included in this study.

#### Response variable

The dependent variable of this study was the cholera outbreak status (death or alive) of Cholera outbreak patients in each region of Ethiopia recorded under EPHI from April 2019 to January 2020.

### Explanatory variables

The selection of explanatory variables is driven by prior research concerning risk factors affecting cholera disease. Previous studies are referenced in creating the variables [5, 6, 8–10]. The explanatory variables were Age of patients, Sex, Admission Status, Dehydration status, another sick person in a family, History of travel, History of contact, Watery Diarrhea, Vomiting, Oral Rehydration Salt (ORS), Intravenous (IV), and Antibiotics. The detail can be found in Table 1.

**Table 1:** Variable description

Variables	Codes	
Cholera outbreak patients	0 = Died	1 = Alive(event)
Age of patients	0 = under 5	1 = 5 to 14
	2 = 15 to 44	3 = 45 and above
Sex	0 = Male	1 = Female
Admission status	0 = Outpatient	1 = Inpatient
Dehydration status	0 = No dehydration	
	1 = Some dehydration 2 = Severe dehydration	
Another sick person in a family	0 = No	1 = Yes
History of travel	0 = No	1 = Yes
History of contact	0 = No	1 = Yes
Watery diarrhea	0 = No	1 = Yes
Vomiting	0 = No	1 = Yes
ORS	0 = No	1 = Yes
IV	0 = No	1 = Yes
Antibiotics	0 = No	1 = Yes

### Statistical models

In this study, the authors applied different statistical methods and used R software for data analysis techniques.

### Bayesian hierarchical logistic regression modeling

Bayesian hierarchical modeling is a statistical model written in multiple levels (hierarchical form) that estimates the parameters of the posterior distribution using the Bayesian method.

The logistic regression model can be changed to linear using the logit link function. And also in a hierarchical model, random coefficient logistic regression is based on linear models for the logit link function that include random effect terms that account for the variation that comes from the groups (regions).

Consider explanatory variables which are a potential explanation for the observed outcomes and denote these variables by ${x}_{1}, {x}_{2},\dots \dots ,{x}_{12}$, these variables were level one (patient’s level) variables. The probability of success (when the outcome of cholera status is Alive) is not necessarily the same for all individuals in a given group (region). Therefore, the success probability depends on the individuals as well as the group is denoted by ${\pi }_{ij}$.

The model is specified by:

1 $${y}_{ij}/{\pi }_{ij}=Ber\left({\pi }_{ij}\right) , {\pi }_{ij}=pr\left({y}_{ij}=1\right)$$

where: ${y}_{ij}=1$ if the patients of cholera status are Alive and 0 if they die. ${\pi }_{ij}$ is the probability of success that i^th^ individual and j^th^ regions presents, for i = 1,2……,n and j = 1,2,…..,11 and ${U}_{0j}$ in equation [3.2] is a random intercept. The probability of success (in our case alive patients) in the logistic regression model can be defined as:

2 $${\pi }_{ij}=\left[\frac{\mathrm{exp}({\beta }_{0}+{{U}_{0j}+\beta }_{1}{x}_{1ij}+... +{\beta }_{12}{x}_{12ij})}{1+\mathrm{exp}({\beta }_{0}+{U}_{0j}{+\beta }_{1}{x}_{1ij}+... +{\beta }_{12}{x1}_{12ij})}\right]$$

The logit link function defines the linear predictor as:

3 $${\eta }_{ij}=logit\left(\frac{{\pi }_{ij}}{1-{\pi }_{ij}}\right)={{\beta }_{0}+{U}_{0j}+\beta }_{1}{x}_{1ij}+ \dots \dots +{\beta }_{12}$$

### Latent Gaussian Models (LGMs)

Latent Gaussian models (LGMs) are a group of models that contains most statistical models used in practice. Indeed, most generalized linear mixed models and generalized additive models that we can perform inference with, are an example of LGM. The R-INLA package is based on the INLA methodology used widely for LGMs.

LGMs represent an important model abstraction for Bayesian inference and include a large proportion, in the sense that the task of statistical inference can be unified for the entire class [11]. The INLA by [11] is focused on providing an approximation of the posterior marginal distribution of the LGMs.

The class of LGMs represented by a hierarchical structure containing three stages. The first stage is formed by the conditional independent likelihood function. The second stage is formed by the latent Gaussian field, where we attribute a Gaussian distribution with mean µ and precision matrix Q to the latent field x conditioned on the hyper parameters θ, and finally, the third stage is formed by prior distribution to the hyper parameters.

Latent Gaussian Model is written as:

4 $$\begin{array}{cc}y/x,{\theta }_{2}\sim \prod_{ij}p\left({y}_{ij}/\eta ,{\theta }_{2} \right)& Likelihood\end{array}$$

5 $$\begin{array}{cc}x/{\theta }_{1}\sim p\left(x/{\theta }_{1}\right)=N\left(0,{Q}^{-1}\right)& Latent field\end{array}$$

6 $$\begin{array}{cc}\theta ={\left[{\theta }_{1},{\theta }_{2}\right]}^{T}\sim p\left(\theta \right)& Hyper-priors\end{array}$$

Considering the LGM, the specific generalized linear mixed model for the outcome of cholera status has the form: $y\sim {\prod }_{ij}p\left({y}_{ij}/{\pi }_{ij}\right)$

$$logit\left({\pi }_{ij}\right)={\beta }_{0}+{b}_{0}+{\beta }_{1}{Age}_{ij}+{\beta }_{2}{Sex}_{ij}+{{\beta }_{3}{Admission\; status}_{ij}+\beta }_{4}{Dehydration\;status}_{ij}+{\beta }_{5}{History\;of\;travel }_{ij}+{\beta }_{6}{History\;of\;contact}_{ij}+{\beta }_{7}{Watery\;diarrhea}_{ij}+{\beta }_{8}{Vomiting}_{ij}+{\beta }_{9}{Other\;sick\;person\;in\;family}_{ij}+{\beta }_{10}{ORS}_{ij}+{\beta }_{11}{IV}_{ij}+{\beta }_{12}{Antibiotics}_{ij}+{U}_{0j}$$

Thus the model is said to be a latent Gaussian model (LGM) if and only if there is a strong assumption that the parameters have joint Gaussian distribution and it can be achieved by assigning Gaussian priors for each element of latent fields. It is to means that x is the joint distribution of the parameters of the linear predictor including it.

7 $$x=\left[\eta , {\beta }_{0}, {{b}_{0}, \beta }_{1}, {\beta }_{2}, {\beta }_{3}, {\beta }_{4}, {\beta }_{5}, {\beta }_{6}, {\beta }_{7}, {\beta }_{8}, {\beta }_{9}, {\beta }_{10},{\beta }_{11},{\beta }_{12} \right]\sim N\left(0,{Q}^{-1}\right)$$

### Integrated Nested Laplace Approximation (INLA)

Bayesians have a full posterior distribution over the possible parameter values and this allows them to get uncertainty of the estimate by integrating the full posterior distribution. The problem with the integration of the denominator in the Bayes formula was intense for the researchers. In the Bayesian approach, Markov Chain Monte Carlo (MCMC) methods were used as a standing point to do practically with the drawback of convergence, very slow in generating sample from the posterior distribution, and Monte Carlo errors [12]. Following the development of Integrated Nested Laplace Approximation (INLA) for Latent Gaussian models (LGMs) in 2009 doing with Bayesian becomes very flexible, accurate, and fast [11].

INLA is the Bayesian statistical inference for latent Gaussian Markov chain Monte Carlo (MCMC), which is the standard tool for inference in such models of Bayesian inference. INLA is specially designed for LGMs. The advantage of the INLA approach over MCMC is that it is much faster and more accurate. MCMC is computationally intensive as compared to INLA [11].

The main goal of the approximation techniques used in the analysis of LGM is to compute posterior marginal for each component of x of expression [5]. Generally, the marginal posterior distribution for each of the parameter vectors can be formulated as:

8 $$\pi \left({x}_{i}/y\right)=\int \pi \left({x}_{i}/\theta ,y\right)\pi \left(\theta /y\right)d\theta$$

In addition, the marginal posterior distribution for each element of hyper-parameter vector:

9 $$\pi \left({\theta }_{i}/y\right)=\int \pi \left(\theta /y\right){d\theta }_{-j}$$

Now, we intended to compute $\pi (\theta /y)$ from which all the relevant $\pi ({\theta }_{i}/y)$ obtained and to determine $\pi ({x}_{i}/\theta ,y)$, which needed to compute the parameter marginal posteriors $\pi ({x}_{i}/y)$.

### Prior distributions of parameters

Bayesian statistical models require prior distributions for all the parameters of the model. Working within the class of LGMs, choosing prior distributions involves choosing priors for all the hyper-parameters θ in the model. Since the latent field is by definition Gaussian.

The R-INLA inbuilt standard priors are the nature of R-INLA packages of INLA function. Different researchers [13–15] briefly used it. According to the study [7] by default, a flat improper prior for the intercept assumed in INLA and all other components of parameters assumed independent Gaussian with mean zero Normal (0,${\sigma }^{2}$) with fixed precision ${\sigma }^{-2}=0.0001$ a priori. If the observation is assumed to follow Bernoulli distribution, by standard the intercept of the model is assigned a Gaussian prior with mean and precision equal to zero and all the fixed parameters assigned zero for mean and 0.001 for precision i.e. N(0, 0.001) priors. Since the researcher assumed a flat prior made the precision was too small and to have a large variance for this prior. The random effect (Region) is Gaussian with zero mean and precision parameters. Then the precision parameter in the random effect is assigned to other distributions of log gamma i.e. log-gamma (1, 0.001).

The other priors are called Penalized Complexity priors, which were developed by [16]. It is imprecise, weakly informative, or strongly informative depending on the way the user tunes an intuitive scaling parameter. Using only weak informative, Penalized Complexity (PC) priors represent a unified prior specification with a clear meaning and interpretation.

### Posterior distribution

The posterior distribution is a way to summarize what we know about uncertain quantities in Bayesian analysis after the data is observed. It is the combination of the prior distribution and the likelihood function.

A great advantage of working in a Bayesian framework is the availability of the entire posterior probability distribution for the parameter(s) of interest. It is always possible and useful to summarize it through some suitable synthetic indicators. The summary statistic typically used is the posterior mean, which, for a hypothetical continuous parameter of interest θ, is:

10 $$E\left(\theta /y\right)={\int }_{\theta \varepsilon\Theta }\theta p\left(\theta /y\right)d\theta$$

where $\Theta$ are all possible values that the variable θ can assume and the integral replaced by sum if θ is discrete.

### Results

Under this section, the authors try to answer the research questions and attain to address the objectives by modeling the data. Here, the descriptive part uses a simple frequency table. In addition, the concept INLA, the results of the models with different fixed and random parameters using two priors. The results obtained from the different models of this study were compared by different criteria.

### Descriptive data analysis

The descriptive statistics were conducted in table 2. There were 81.61% of patients are survived from cholera outbreak disease and the rest 18.39% have died. Of those female patients are 44.95% and 55.05% are male patients in Ethiopia in the study period. The age group under five, between 5 and 14, between 15 and 44, and above 45 were 13.26%, 19.10%, 52.98%, and 14.66% respectively. There were 17.02% of patients were treated by ORS, about 38.06% were treated by IV, and 68.67% of patients were treated with antibiotics. (Refer to table 2 in Additional file 1: appendix I).

**Fig. 1:**
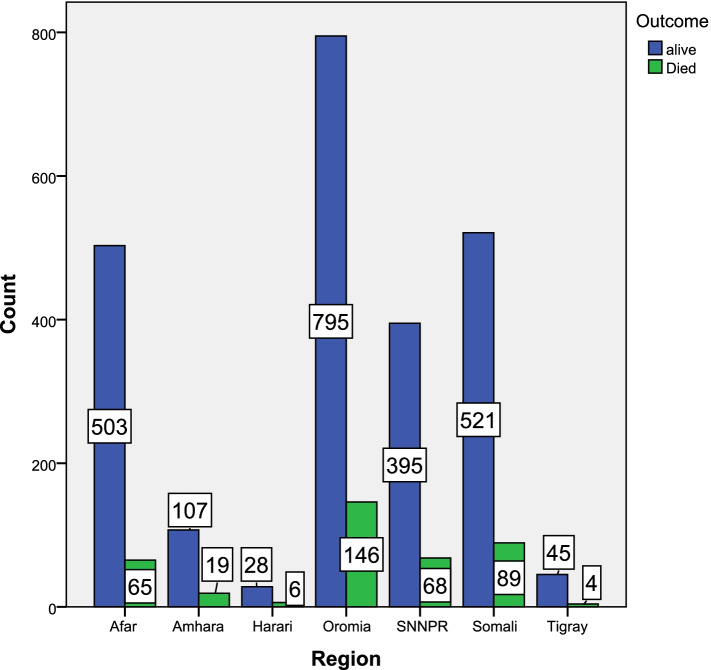
Cholera status across region

Figure 1 shows most of patients 795 from Oromia region was alive and the rest 146 were died. Following Oromia region 503 patients from Afar were alive and 65 patients were died. Around 28 patients were alive from Harari and 6 patients were died.

**Fig. 2:**
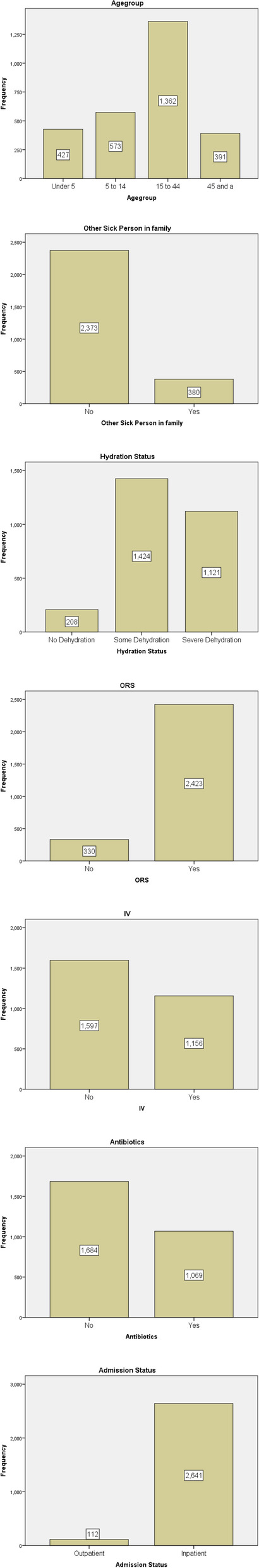
Bar chart for significant variables

There were 2373 patients doesn’t have other sick person in a family and 380 patients have other sick person in a family. The dehydration status of patients for not dehydrated, some dehydrated and severe dehydrated were 208, 1424 and 1121 respectively. Admission status of patients shows the admission statuses of 2641 patients were inpatients and 112 were outpatients (Fig. 2).

### Model-based data analysis

The intercept-only model helps to see the average cholera case in the absence of covariates and to see its variability across the regions in Table 3. It indicated that keeping all the factors to be constant, the average number of cholera cases in Ethiopia is about 5.458 without considering the regional variability. On the other hand, there was 39% variation across the region in Ethiopia (1/2.58 = 0.39). This is determined by considering the mathematical relationship between precision and variance that one is the inverse of the other (Refer to table 3 in the Additional file 1: appendix).

Table 4 below is the final model summary of a full model with R-INLA inbuilt standard prior and incorporating the variation across the region. For, an easy understanding of the interpretation, the researcher relies on interpreting the odds of each coefficient. Keeping all the categorical factors at their reference category, the odds of surviving from cholera disease is about 7.645 (Refer to table 4 in the Additional file 1: appendix).

With the data under this study and techniques applied, since the 95% CI for exp **(**$\widehat{\beta }$**)** include one there is not enough evidence that supports the significance of factors like gender, age (5 to 14), age (above 45), History of travel, History of contact person, watery diarrhea, and vomiting. On the other hand, other variables include one; there is enough evidence that supports the significance of factors like admission status, age group 15 to 44, another sick person in a family, some dehydration, severe dehydration, ORS, IV, and antibiotics (Refer to table 4 in the Additional file 1: appendix).

The risk factor for admission status is significant and the odds of surviving from cholera outbreak disease for that inpatient status are 0.609 times less than outpatient status. This is because the inpatient is often those are at intensive sickness and they may have low probability to survive than those who are not admitted to staying at the health center. (Refer to table 4 in the Additional file 1: appendix).

The odds of surviving from cholera disease in those aged between 15 and 44 is about 1.549 times more than those aged under 5 years. The risk factor that asks whether there was a sick person in the family is also significant and the odds of surviving after being caught by cholera disease for those who have a sick person in their family is about 0.758 times less than those who have no such history. This is mean that if there is a person that already has cholera disease in the family, there is a high probability that the other can also develop which leads them also to have less chance to survive (Refer to table 4 in the Additional file 1: appendix).

The other significant potential determinant for cholera status is dehydration status. It generally revealed that higher dehydration status has less chance to survive from the disease. The odds of surviving after having cholera for those with some dehydration status and severe dehydration status are 0.571 and 0.399 times less than no dehydration problem respectively. This is just scientific to say that the more problem of dehydration, there is less chance to survive from any disease (Refer to table 4 in the Additional file 1: appendix).

The treatment factors (ORS, IV, and antibiotics) are significant. The odds of surviving after having cholera for those who take the treatment ORS are 1.579 times more than those who have not taken the treatment. The odds of surviving after having cholera for those who take IV and antibiotics were 1.608, and 1.624 more than those who have not taken the treatments. At the same time, it also shows that antibiotic treatment seems slightly better. There is a 16% variability of cholera disease across the regions of Ethiopia is 0.16 (1/6.35) (Refer to table 4 in the Additional file 1: appendix I).

The table also presents the median and mode of the posterior distribution. Those values for all the factors are almost the same as the mean of the posterior distribution. Hence, this leads us to say that the distribution is approximately symmetric. Further, evidence to assure the symmetry is that the value of Kullback–Leibler divergence (KLD) is zero for all factors which are to means that the posterior distribution is well approximated by a Normal distribution and is symmetry (Refer to table 4 in the Additional file 1: appendix).

### Model comparison

The most typically used to measure model fit based on the deviance for Bayesian models is Deviance Information Criterion (DIC). It is an overview of the Akaike-information criterion (AIC) developed particularly for Bayesian model comparison and it is the sum of two components, likewise Watanabe-Akaike information criterion (WAIC) is generalized version of AIC and Bayesian information criterion (BIC) works in singular models.

WAIC has the desirable property of averaging over the posterior distribution rather than conditioning on a point estimate and does not rely on posterior means of parameters compared to DIC.

Model comparison is important to choose the best model; in this study, the researcher compares the model using two deviances. Therefore, we have four models:

Model 1: LGM with intercept only model under standard priors, Model 2: LGM with covariates of fixed effects only, Model 3: LGM including covariates of both fixed and random effects with standard priors, and Model 4: LGM including covariates of both fixed and random effects with PC priors.

For Bayesian model selection, the Deviance Information Criterion (DIC) is a hierarchical modeling generalization of the Akaike information criterion is used. The lowest expected deviance has a higher posterior probability, which we can say better fit the data. The same is true for Watanabe-Akaike Information Criterion (WAIC).

Table 5 is the summary of DIC and WAIC for four models under different parameters (different priors). Model 3 has small value of DIC (2531.33) and WAIC (2531.71) compared to the other models. Then model 3 better fit the data relative to the other three models (model 1, model 2, and model 4). The authors were able to compare the same model under different priors because it helps to avoid the problem of model fit due to bad priors and also used for further investigation as for whether the recent informative PC priors was more efficient than the R-INLA inbuilt standard priors or not. (Refer to table 5 in the Additional file 1: appendix I).

Considering the above evidence (model comparison technique), we selected LGM of Bernoulli distributional assumption of cholera outbreak patients including covariates of fixed and random effects under standard priors as a better model.

### Model-checking

The numerical problems may occur in the predictive measure when the CPO and PIT indexes are computed. The R-INLA provides automatically a failure vector that contains 0 or 1 value for each observation, a value equal to 1 indicates that for the failure vector. For this study since the sum of failure in CPO from the fitted model was 0, no failure has been detected and then we can conclude that no numerical problems were occurring in the predictive measure.

**Fig. 3:**
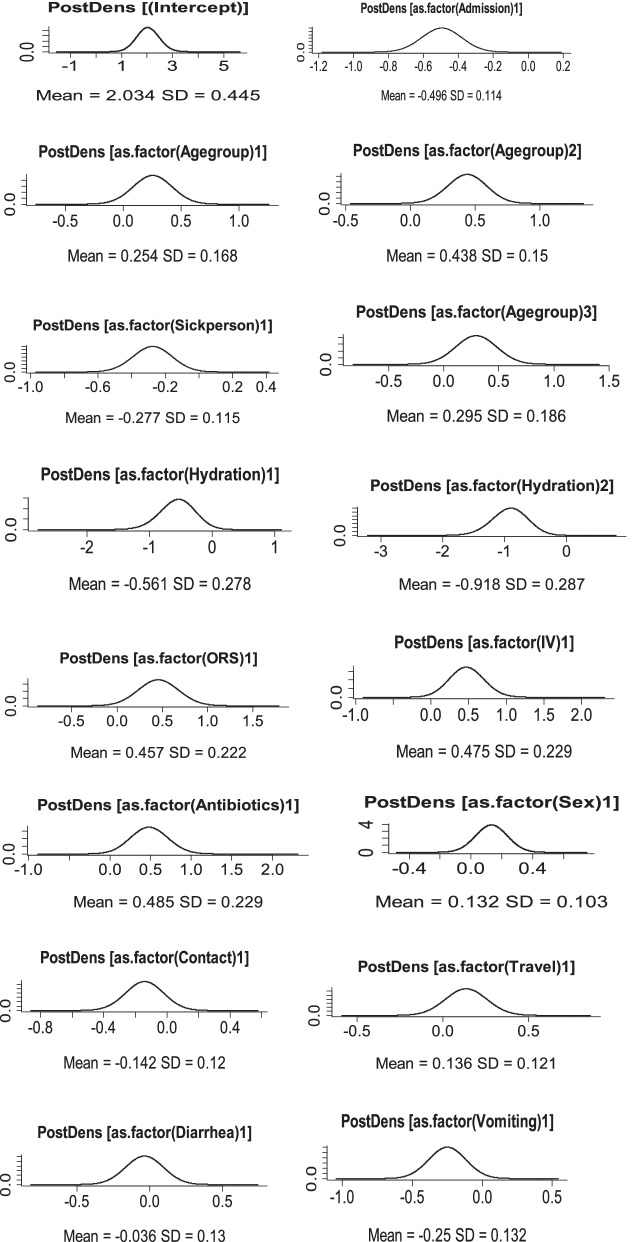
Density plots for each categorical variable

**Fig. 4:**
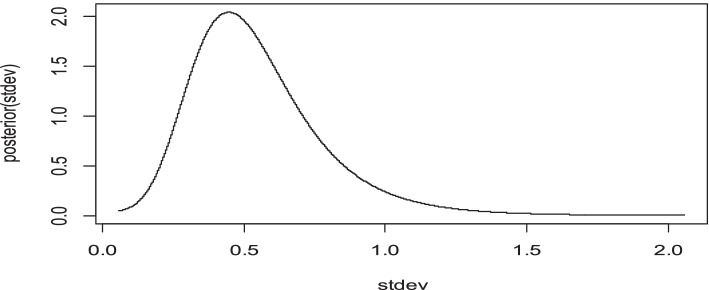
Posterior marginal distribution of standard deviation for the random effects

Figure 3 shows the posterior distribution of those variables was approximated by the normal distribution. Since density plot is the usual measure of convergence in the Bayesian approach, we used this technique to see the convergence of the estimated parameters. Whereas, the posterior marginal distribution of standard deviation for the random effects is right skewed as expected (Fig. 4).

### Discussion

The number of male patients with cholera disease 55.05% was greater than the number of female patients with the same disease. These results were linked with [4] which also presents that the number of males was more affected than females in Ethiopia. Likewise, the number of cholera patients in the age group 15–44 years was greater than the three age groups (less than 5, 5 to 14, and 45 and above). These results also related with the same study, those who were between the ages 15 to 44 years were more affected than the three categories of age group (less than 5, 5 to 14 and 45 and above).

The LGM with approximation technique of INLA is efficient and the effectiveness and importance of the model helped by the study [17]. The significant variables in this study were related to the study [18].

Likewise, history of travel and history of contact doesn’t have a significant effect on cholera status but in another study [19] travel history and contacts, they found that traveling to another governorate having had contact with a potential cholera case were significantly associated with being a case.

The cholera patients of age group 15 to 44 years have higher odds of surviving from the disease than those aged under 5. Then in the other study [8], the cholera disease affected all age groups, age group 5–9 years had the highest proportion of cases excluded aged 0–5 years. On the other hand, dehydration status has a significant effect on cholera disease; the higher dehydration status indicates a less chance to survive from the disease. This result is associated with the study [18].

The factor of another sick person in the family was a significant effect on cholera status this means, if there is a person that already has cholera disease in the family, there is a high probability that the other can also develop the disease and have less chance survive. This variable was also significant in the study [18]. The treatment factors (ORS, IV, and antibiotics) were a significant effect on cholera status. This means people who take the three treatments have a better chance of survival from the disease as compared to those who have not taken the treatment. This result is also reliable with the same study [18].

The random effect of this study was significant and varies across regions and this indicates that including regions as random effect are important. Therefore, the Oromia region was the most affected compared with the other regions. There was a study that identifies cholera disease varies across geographic variations [4].

The model comparison was used by using DIC and WAIC, then four models were compared to choose the best model. The result of DIC and WAIC indicated that model 2 which was the LGM of Bernoulli distributional assumption with fixed effects only was better than model 1. The effects of the priors, model 3 which were the LGM of Bernoulli distributional assumption with fixed and random effects with standard priors was better than model 4. Finally, model 3 was selected comparative best model to fit cholera status in Ethiopia. This comparison was helped by the study [11, 17].

For model checking, CPO and PIT were used in this study. The numerical problem may occur during the computation of CPO and PIT. In R-INLA the failure vector which contains 0 or 1, 1 indicates for the corresponding observation the predictive measures are not reliable due to some problems. In this study the sum of the number of failures in CPO was zero, no failure was detected and meaning that no numerical problem has occurred. This model checking was also used in the study [20].

## Conclusion

The study aimed to identify risk factors associated with cholera disease in Ethiopia using the Bayesian hierarchical model, cholera disease status as response variable as alive or dead. There were 81.61% of patients are survived from cholera outbreak disease and the rest 18.39% have died.

The LGM indicated the predictor variables were sex, age, admission status, history of travel, history of contact, another sick person in the family, dehydration status, watery diarrhea vomiting, ORS, IV, and antibiotics were significantly associated with cholera outbreak disease. Using DIC and WAIC, the LGM of Bernoulli distributional assumption of cholera status including fixed and random effects using standard priors has been selected as the best model fit the data well.

Based on the significant covariates, interested researchers may validate by applying it to another data so that finally it can be used as important impute for the policy makers. All family members should give attention to the disease, and health centers should give awareness of cholera disease across all regions. Further research may add some important variables to get more significant variables and assess the spatial epidemiology of cholera disease in Ethiopia to identify the hotspot of cholera disease.

## Supplementary Information

**Additional file 1: Appendix I.**

## Data Availability

Any interested person or researcher can contact the first author with the corresponding email, if they want to request the data used in this study.

## Abbreviations

CI: Credible Interval
CPO: Conditional Predictive Ordinate
DIC: Deviance Information Criterion
EPHI: Ethiopia Public Health Institute
INLA: Integrated Nested Laplace Approximation
IV: Intravenous
KLD: Kullback–Leibler divergence
LGM: Latent Gaussian Model
MCMC: Markov Chain Monte Carlo
ORS: Oral Rehydration Salt
PIT: Probability integral transform
SNNPR: Southern Nations Nationalities and Peoples’ Region
WAIC: Watanabe-Akaike Information Criterion
WHO: World Health Organization
