# Supplementary material for: Determining factors associated with cholera disease in Ethiopia using Bayesian hierarchical modeling
Source: BMC Public Health. 2022 Sep 20;22:1779. doi: 10.1186/s12889-022-14153-1 (PMC9487065; doi:10.1186/s12889-022-14153-1)
Supplement: Supplementary file 1 — Additional file 1: Appendix I. [file 12889_2022_14153_MOESM1_ESM.docx]

**Appendixes**

**Appendix I: Tables**

Table 2: Frequency for socio-demographic, cholera outbreak and Treatments

| Variables | Categories | Frequency(percent) |
| --- | --- | --- |
| Cholera outbreak | Alive(survived) | 2277(81.61) |
|  | Death | 513(18.39) |
| Sex of patients | Female | 1254(44.95) |
|  | Male | 1536(55.05) |
| Age group | Under 5 | 370(13.26) |
|  | 5 to 14 | 533(19.10) |
|  | 15 to 44 | 1478(52.98) |
|  | Above 45 | 409(14.66) |
| ORS | Yes | 475(17.02) |
|  | No | 2315(82.98) |
| IV | Yes | 1062(38.06) |
|  | No | 1728(61.94) |
| Antibiotics | Yes | 1916(68.67) |
|  | No | 874(31.33) |

Table 3: Intercept only model

| Fixed effects: | Posterior Mean ($\hat{\boldsymbol{\beta}}$) | | St. Dev | Exp ($\hat{\boldsymbol{\beta}}$) | | 95% CI for exp($\hat{\boldsymbol{\beta}}$) | kld |  |
| --- | --- | --- | --- | --- | --- | --- | --- | --- |
| Intercept | 1.697 | | 0.297 | 5.458 | | (3.086, 10.278) | 0.001 |  |
|  | | Model hyper parameters: | | | | | | |
| Precision for Region | 2.58 | | 2.10 | | - | (0.496, 7.58) | -- |  |

Table 4: Full model with standard priors

| Fixed effects: | Posterior Mean ($\hat{\boldsymbol{\beta}}$) | St .Dev | exp ($\hat{\boldsymbol{\beta}}$) | 95% CI for exp ($\hat{\boldsymbol{\beta}}$) | Kld |
| --- | --- | --- | --- | --- | --- |
| Intercept | 2.034 | 0.445 | 7.645 | (3.215,18.523) | 0 |
| Sex (Female) | 0.132 | 0.103 | 1.141 | (0.933,1.397) | 0 |
| Admission (Inpatient)* | -0.496 | 0.114 | 0.609 | (0.486,0.761) | 0 |
| Age (5 to 14) | 0.254 | 0.168 | 1.289 | (0.927,1.791) | 0 |
| Age (15 to 44)* | 0.438 | 0.150 | 1.549 | (1.151,2.077) | 0 |
| Age (above 45) | 0.283 | 0.184 | 1.327 | (0.925,1.908) | 0 |
| History of travel (Yes) | 0.136 | 0.121 | 1.146 | (0.333,1.454) | 0 |
| History of contact (Yes) | -0.142 | 0.120 | 0.868 | (0.686,1.096) | 0 |
| Another sick person in family* | -0.277 | 0.115 | 0.758 | (0.604,0.950) | 0 |
| Dehydration status (Some)* | -0.561 | 0.278 | 0.571 | (0.324,0.964) | 0 |
| Dehydration status (Severe)* | -0.918 | 0.287 | 0.399 | (0.223,0.688) | 0 |
| Watery diarrhea (Yes) | -0.036 | 0.130 | 0.965 | (0.747,1.246) | 0 |
| Vomiting (Yes) | -0.250 | 0.132 | 0.779 | (0.600,1.009) | 0 |
| ORS (Yes)* | 0.457 | 0.222 | 1.579 | (1.025,2.447) | 0 |
| IV (Yes)* | 0.475 | 0.229 | 1.608 | (1.035,2.537) | 0 |
| Antibiotics (Yes)* | 0.486 | 0.229 | 1.624 | (1.043,2.560) | 0 |
| Results of Random effects | | | | |  |
| Precision for random term | 6.35 | 11.80 | - | (0.778,25.48) |  |

*Indicate significant variables

Table 5: Model comparison using WAIC, DIC, and effective number of parameters

| Models | DIC | WAIC |
| --- | --- | --- |
| Model 1 | 2584.45 | 2584.11 |
| Model 2 | 2561.57 | 2562.18 |
| Model 3 | 2531.33 | 2531.71 |
| Model 4 | 2549.69 | 2550.03 |

**Appendix II: Figures**


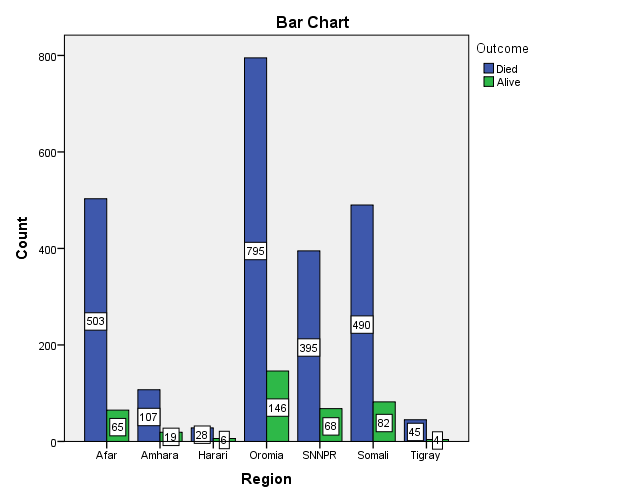


Figure 1: Cholera status across region


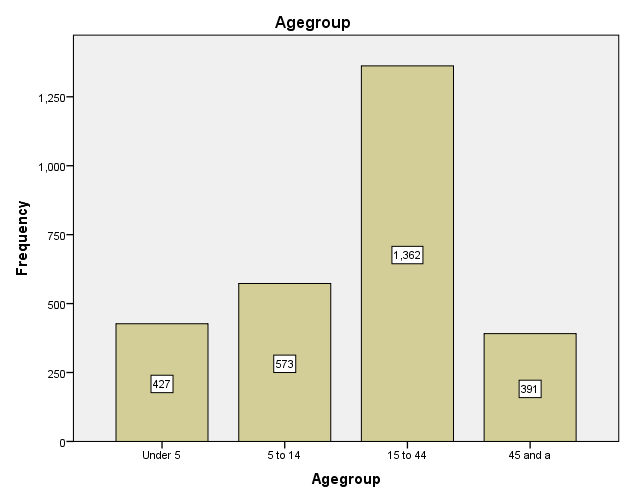


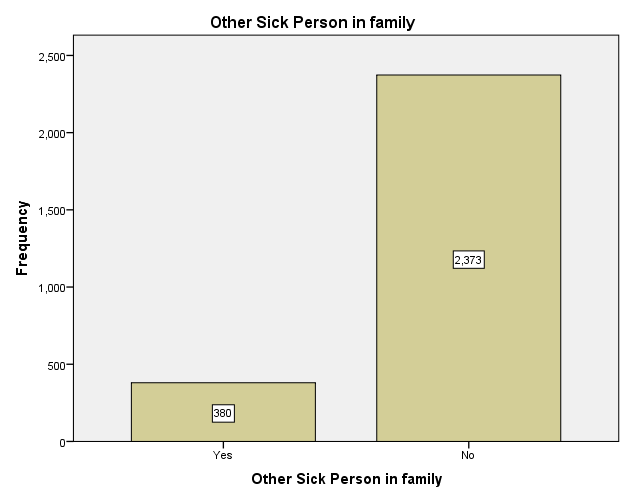


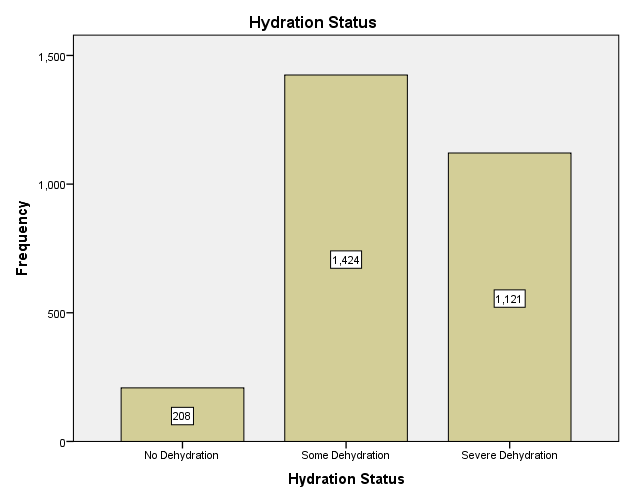


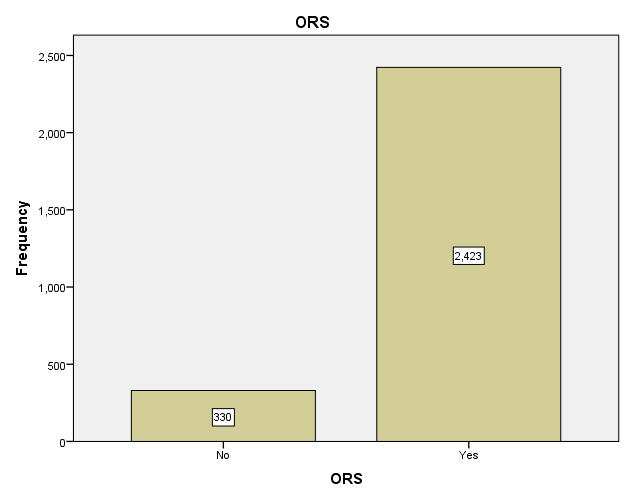


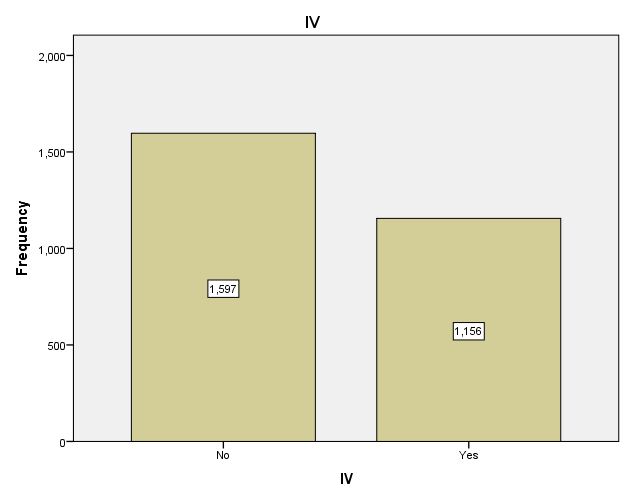


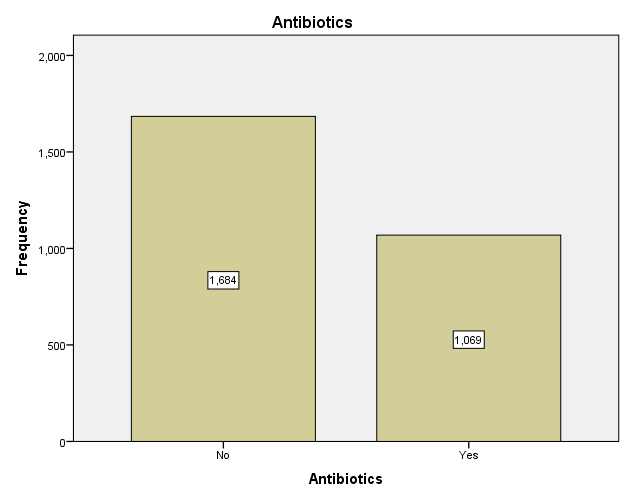


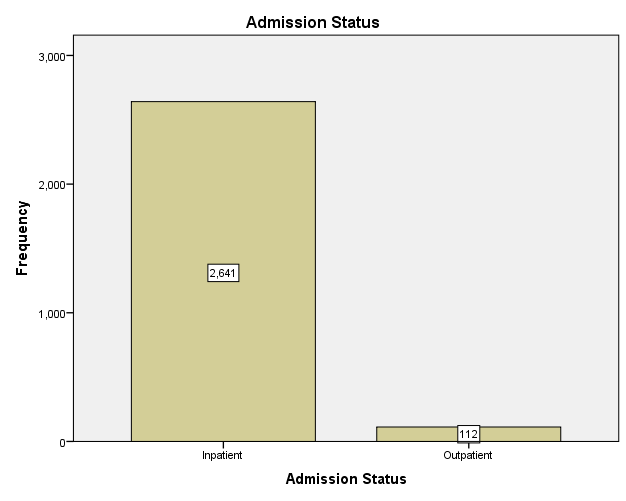


Figure 2: Bar chart for Significant Variables

Figure 3: Density plots for each categorical variable

Figure 4: Posterior marginal distribution of standard deviation for the random effects
